# Comparative Efficacy and Safety of Netarsudil-Containing Interventions for Intraocular Pressure Control: A Systematic Review and Network Meta-Analysis

**DOI:** 10.7759/cureus.78432

**Published:** 2025-02-03

**Authors:** Bakhtawar Awan, Mohamed Elsaigh, Areej Tariq, Mohammed Badee

**Affiliations:** 1 General Surgery, Northwick Park Hospital, London, GBR; 2 General and Emergency Surgery, Northwick Park Hospital, London North West University, London, GBR; 3 Opthalmology Department, Shaikh Zayed Hospital, Lahore, PAK; 4 Vitreoretinal Surgery, Perfect Vision Eye Hospital, Cairo, EGY

**Keywords:** intraocular pressure (iop), netarsudil, network meta-analysis, ophthalmology, systematic review

## Abstract

Netarsudil has been approved for lowering elevated intraocular pressure (IOP), showing effectiveness through two distinct mechanisms. It is also effective when used in combination with other therapies to enhance outcomes. This study aims to compare the drug’s effectiveness with other treatments, both as a standalone and in combination therapies, while also assessing potential adverse effects to evaluate its overall safety and suitability. We systematically searched PubMed, Cochrane, Web of Science, and Scopus till the 7th of October. Data from eligible studies were extracted and combined using a frequentist network meta-analysis, presented as mean differences (MDs) for continuous outcomes and risk ratios (RRs) along with their 95% confidence intervals (CIs). We used the Cochrane risk-of-bias (ROB) tool to assess the quality of the included RCTs. Netarsudil 0.02%/latanoprost 0.005% fixed-dose combination (FDC) q.d. was the most effective in reducing IOP in one-, two-, and six-week follow-ups in addition to the three-month follow-up. The netarsudil-containing medication was associated with higher adverse events compared to other arms. Netarsudil 0.02%/latanoprost 0.005% FDC q.d. and bimatoprost 0.03%/timolol 0.5% FDC emerged as the most effective therapies for lowering IOP, with each showing significant advantages at different follow-up points. Both FDCs achieved substantial reductions in IOP and a high proportion of patients reaching target IOPs. However, safety profiles indicate that traditional therapies like latanoprost 0.005% and timolol 0.5% may have fewer side effects, including lower incidences of blurred vision, conjunctival hemorrhage, and conjunctival hyperemia.

## Introduction and background

Glaucoma represents a spectrum of ocular pathologies that result in progressive optic neuropathy, compromising the integrity of the neural pathway essential for transmitting visual information from retinal photoreceptors to cortical processing centers [1].

Optic nerve damage is commonly linked to elevated intraocular pressure (IOP); however, glaucoma can also develop in people with normal eye pressure. This condition is not confined to a particular age group and affects individuals of all ages. Although it is more common in older adults, glaucoma is a leading cause of blindness, especially in those aged 60 and above [2].

Glaucoma is categorized into several types, with primary open-angle glaucoma being the most common, along with other forms, including secondary glaucoma. Early detection and treatment are crucial, as they can help protect vision and slow the progression of the disease [3].

Netarsudil is a medication primarily used to treat glaucoma and ocular hypertension, commonly administered as eye drops. It helps lower IOP in the eyes, which is essential for managing these conditions. Netarsudil functions by inhibiting the Rho kinase (ROCK) pathway, leading to a reduction in IOP. Beyond its main effect, ROCK inhibitors like netarsudil offer additional benefits. These include enhancing the function of the trabecular meshwork, improving retinal blood flow, protecting neurons from various stressors, and aiding in wound healing. These added effects could make netarsudil particularly valuable in glaucoma treatment [4]. 

The ROCK signaling pathway plays a key role in multiple cellular processes, including cell proliferation, cytoskeletal remodeling, and cell adhesion. It is activated by various bioactive factors present in the aqueous humor. Research suggests that the ROCK pathway may be essential for corneal endothelial cell function in both healthy and disease states. In addition, it has been recognized as a critical regulator of fluid outflow through the trabecular meshwork, a function that is impaired in people with glaucoma [5].

This systematic review and network meta-analysis aims to evaluate the safety and efficacy of netarsudil, whether used as monotherapy or combined with other IOP-lowering medications.

## Review

Methods

The methodology of our research adhered to the standards outlined in the Cochrane Handbook for Systematic Reviews of Interventions. Our findings were then documented following PRISMA (Preferred Reporting Items for Systematic Reviews and Meta-Analyses) guidelines [6,7].

Search Strategy and Data Collection

We searched PubMed, Cochrane, Web of Science, and Scopus using these terms ("Open-angle glaucoma" OR POAG OR "Ocular Hypertension" OR "Intraocular Pressure" OR "Exfoliation glaucoma" OR pseudoexfoliation OR "Corneal Edema" OR "Compensated Glaucoma" OR "Open Angle Glaucomas" OR "Pigmentary Glaucoma" OR "simple Glaucoma" OR "Compensative Glaucoma" OR "Primary Open Angle Glaucoma" OR "Secondary Open Angle Glaucoma") AND (Netarsudil OR "AR-13324" OR Rhopressa OR Rhokiinsa OR Rocklatan).

Selection Criteria

We included RCT studies that followed the criteria of netarsudil or latanoprost or latanoprostene or timolol in open-angle glaucoma patients. We used EndNote (Clarivate, UK) for the removal of duplications.

Data Extraction

We extracted the data that presented the baseline and summary of the included studies, such as study ID, group of interventions, number of population, gender, age, study eye diagnosis, race, mean time since diagnosis, mean diurnal IOP (mmHg)at baseline, and conclusion. All the data were extracted using Excel sheets (Microsoft Corp., USA). 

Quality Assessment

We used the Cochrane risk-of-bias (ROB) tool (version 1) to assess the quality of the included studies. We employed the evaluation framework outlined in Section 8.5 of the Cochrane Handbook (version 5.1.0), which examines seven components of methodological quality. These components include the generation and concealment of allocation sequences, blinding procedures for both participants/personnel and outcome assessors, handling of incomplete data, selective reporting, and additional sources of bias. Following the handbook's quality assessment guidelines in Part 2, Chapter 8.5, we categorized each component as having either low, unclear, or high ROB [8].

Statistical Analysis

Statistical analyses were conducted utilizing MetaInsight version 3.14, a web-based platform integrating R-shiny and netmeta packages for network meta-analyses. We implemented random-effects frequentist network meta-analysis, where continuous outcomes were synthesized using mean differences (MDs) with corresponding 95% confidence intervals (CIs). By contrast, dichotomous outcomes were analyzed through risk ratios (RRs). Heterogeneity assessment encompassed clinical, methodological, and statistical dimensions, with statistical heterogeneity specifically quantified via I² statistics [9].

Results

**Figure 1:**
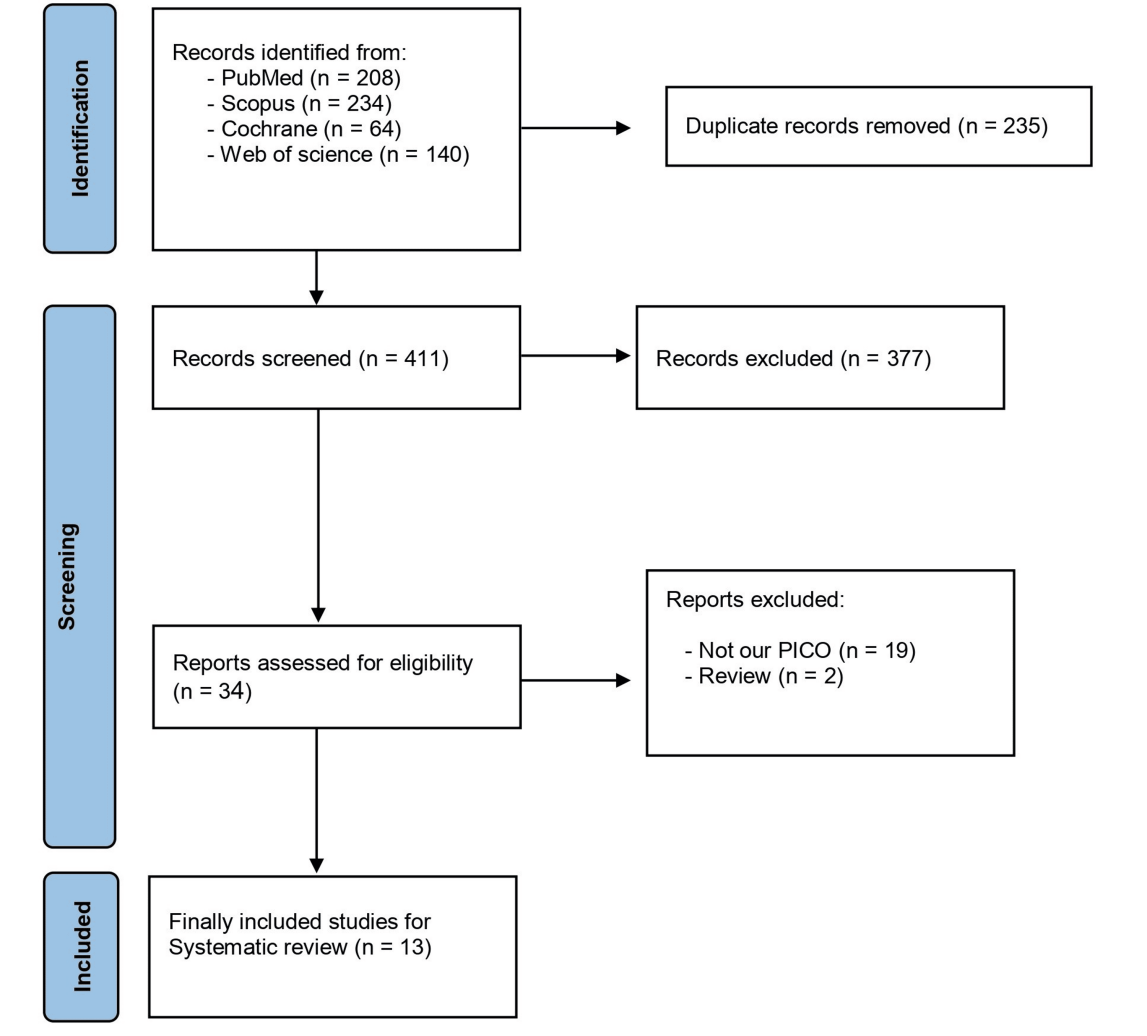
PRISMA (Preferred Reporting Items for Systematic Reviews and Meta-Analyses) flow diagram References: [10-22]

The initial search across four databases yielded 695 citations. After removing 225 duplicate records using EndNote, 470 articles remained for title and abstract screening. Following this screening process, 13 [10-22] studies met the inclusion criteria and were selected for the final analysis. The full PRISMA is presented in Figure 1.

The included population's baseline characteristics and included studies' summary are shown in Table 1. 

**Table 1:** Baseline and summary charactaristics of the included papers. NA: not available, SD: standard deviation, IOP: intraocular pressure, AEs: adverse events, NET: netarsudil, LAT: latanoprost, FDC: fixed-dose combination, BIM: bimatoprost, OHT: ocular hypertension, POAG: primary open-angle glaucoma References: [10-20]

Study ID	Groups	N	Female, N (%)	Mean age, yrs (SD)	Age category, n (%)		Study eye diagnosis, n (%)		Race, N (%)							Mean time since diagnosis, weeks (SD)	Mean diurnal IOP(mmHg)at baseline Mean (SD)	Conclusion
					<65 years	>=65 years	Ocular hypertension	Open-angle glaucoma	Asian	Black or African American	White	Caucasian	Native American	Other	Multiple			
Araie, 2023 [10]	Netarsudil	122	78 (63.9)	61.4 (13.18)	63(51.6)	59(48.4)	32(26.2)	90(73.8)	NA	NA	NA	NA	NA	NA	NA	NA	20.48 (2.77)	Netarsudil 0.02% was superior to ripasudil 0.4% in the reduction of IOP following four weeks of treatment, and was generally well-tolerated and showed a favorable safety profile in Japanese subjects. Netarsudil ophthalmic solution 0.02% QD could provide an important new treatment for patients with POAG or OHT with its clinically significant IOP reduction as a second-generation ROCK inhibitor.
	Ripasudil	123	66(53.7)	62.0 (12.99)	60(48.8)	63(51.2)	35(28.5)	88(71.5)	NA	NA	NA	NA	NA	NA	NA	NA	20.83 (3.55)	
Araie, 2021 [11]	Netarsudil 0.01%	55	36(65.5)	62.7 (14.6)	22(40.0)	33(60.0)	16(29.1)	39(70.9)	NA	NA	NA	NA	NA	NA	NA	NA	20.51 (2.84)	Netarsudil ophthalmic solutions 0.01%, 0.02%, and 0.04% dosed QD (P.M.) demonstrated superiority to placebo in terms of hypotensive effectiveness at week 4 and were found to be safe and generally well tolerated. Netarsudil 0.02% QD provided an optimal efficacy and safety profile for the treatment of Japanese patients with POAG or OHT.
	Netarsudil 0.02%	54	29(53.7)	64.1 (12.2)	23(42.6)	31(57.4)	15(27.8)	39(72.2)	NA	NA	NA	NA	NA	NA	NA	NA	20.28 (2.80)	
	Netarsudil 0.04%	52	27(52.9)	62.0 (13.6)	28(54.9)	23(45.1)	16(31.4)	35(68.6)	NA	NA	NA	NA	NA	NA	NA	NA	20.76 (3.20)	
	Placebo	55	31(56.4)	64.6 (12.6)	23(41.8)	32(58.2)	15(27.3)	40(72.7)	NA	NA	NA	NA	NA	NA	NA	NA	21.14 (3.70)	
Kahook, 2019 [12]	Netarsudil 0.02% q.d.	251	148 (59.0)	65.3 (11.48)	111 (44.2)	140 (55.8)	NA	NA	2 (0.8)	69 (27.5)	178 (70.9)	NA	2 (0.8)	0	0	NA	NA	Dosing of netarsudil 0.02% was effective, consistently lowering IOP through 12 months, and was tolerated by the majority of patients.
	Netarsudil 0.02% b.i.d.	254	165 (65.0)	64.1 (12.46)	126 (49.6)	128 (50.4)	NA	NA	6 (2.4)	69 (27.2)	177 (69.7)	NA	0	1 (0.4)	1 (0.4)	NA	NA	
	Timolol 0.5% b.i.d.	251	150 (59.8)	63 (11.81)	131 (52.2)	120 (47.8)	NA	NA	6 (2.4)	76 (30.3)	166 (66.1)	NA	0	1 (0.4)	2 (0.8)	NA	NA	
Khouri, 2019 [13]	Netarsudil 0.02%	351	208 (59.3)	64.1 (11.6)	165 (47.0)	186 (53.0)	NA	NA	7 (2.0)	84 (23.9)	259 (73.8)	NA	NA	1 (0.3)	NA	364.1 (367.3)	NA	Netarsudil QD (PM), a first-in-class IOP-lowering medication, was noninferior to timolol BID and was associated with tolerable ocular AEs.
	Timolol 0.5%	357	237 (66.4)	64.5 (11.0)	164 (45.9)	193 (54.1)	NA	NA	6 (1.7)	75 (21.0)	274 (76.8)	NA	NA	2 (0.6)	NA	344.2 (341.1)	NA	
Lewis, 2016 [14]	PG324 0.01%	74	47 (63.5)	65.4 (11.26)	29 (39.2)	45 (60.8)	NA	NA	3 ( 4.1)	15 (20.3)	0	56 (75.7)	0	NA	NA	NA	NA	In this study, the fixed-dose combination of AR-13324 0.02% and latanoprost 0.005% in PG324 Ophthalmic Solution provides clinically and statistically superior ocular hypotensive efficacy relative to its individual active components at the same concentrations. The only safety finding of note was transient asymptomatic conjunctival hyperaemia, which was typically of mild severity.
	PG324 0.02%	73	39 (53.4)	64.2 (11.07)	35 (47.9)	38 (52.1)	NA	NA	1 ( 1.4)	10 (13.7)	0	62 (84.9)	0	NA	NA	NA	NA	
	Latanoprost 0.005%	73	46 (63.0)	65.1 (12.80)	28 (38.4)	45 (61.6)	NA	NA	1 ( 1.4)	12 (16.4)	0	60 (82.2)	0	NA	NA	NA	NA	
	AR-13324 0.02%	78	43 (55.1)	64.8 ( 11.28)	33 (42.3)	45 (57.7)	NA	NA	2 ( 2.6)	17 (21.8)	0	58 (74.4)	1 ( 1.3)	NA	NA	NA	NA	
Price, 2020 [15]	Netarsudil	95	54 (57)	median (range)	NA	NA	NA	NA	NA	NA	NA	NA	NA	NA	NA	NA	NA	Netarsudil did not produce a statistically significant reduction in the risk of steroid-induced IOP elevation after corneal transplantation relative to placebo.
	Placebo	96	61 (64)	median (range)	NA	NA	NA	NA	NA	NA	NA	NA	NA	NA	NA	NA	NA	
Serle, 2018 [16]	Netarsudil 0.02% q.d	251	148 (59.0)	65.3 (11.48)	111 (44.2)	140 (55.8)	84 (33.5)	167 (66.5)	2 (0.8)	69 (27.5)	178 (70.9)	NA	2 (0.8)	0	0	NA	NA	Once-daily dosing of netarsudil 0.02% was found to be effective and well-tolerated for the treatment of patients with ocular hypertension and open-angle glaucoma. It is suggested that the drug may be a useful addition to the armamentarium of ocular hypotensive medications.
	Netarsudil 0.02% b.i.d.	254	165 (65.0)	64.1 (12.46)	126 (49.6)	128 (50.4)	96 (37.8)	158 (62.2)	6 (2.4)	69 (27.5)	177 (69.7)	NA	0	1 (0.4)	1 (0.4)	NA	NA	
	Timolol 0.5% b.i.d.	251	150 (59.8)	63 (11.81)	131 (52.2)	120 (47.8)	80 (31.9)	171 (68.1)	6 (2.4)	76 (30.3)	166 (66.1)	NA	0	1 (0.4)	2 (0.8)	NA	NA	
Shahid, 2023 [17]	Netarsudil 0.02%	34	20	44.68 (10.32)	NA	NA	NA	NA	NA	NA	NA	NA	NA	NA	NA	NA	NA	The study found meaningful IOP reduction with netarsudil monotherapy and combination therapy as compared to prostaglandin analogs. Therefore, IOP‑lowering effect of netarsudil 0.02% is non‑inferior to bimatoprost 0.01% in patients with POAG and ocular hypertension. Netarsudil can be used both as monotherapy and combination therapy. In addition, it can be a useful adjunct in patients who are on maximal glaucoma therapy. Although conjunctival hyperemia was reported with a higher frequency and severity, it should not be a deterrent to its routine use in glaucoma management.
	Bimatoprost 0.01%	32	15	51.75 (10.85) )	NA	NA	NA	NA	NA	NA	NA	NA	NA	NA	NA	NA	NA	
	combination therapy	34	15	50.07 (18.75	NA	NA	NA	NA	NA	NA	NA	NA	NA	NA	NA	NA	NA	
Stalman, 2024 [18]	NET/LAT FDC	218	131 (60.1)	67.3 (12.03)	NA	147 (67.4)	94 (43.1)	124 (56.9)	0	4 (1.8)	210 (96.3)	NA	NA	4 (1.8)	NA	NA	25.10 (3.41)	Once-daily NET/LAT was non-inferior to BIM/TIM in IOP reduction in OAG and OHT, with AEs consistent with previous findings. NET/LAT offers a compelling alternative FDC treatment option for OAG and OHT.
	BIM/TIM FDC	212	92 (43.4)	67.0 (11.27)	NA	133 (62.7)	100 (47.2)	112 (52.8)	3 (1.4)	5 (2.4)	200 (94.3)	NA	NA	4 (1.9)	NA	NA	24.81 (3.26)	
Walters, 2019 [19]	Netarsudil/Latanoprost FDC	245	152 (62.0)	64.2 (11.81)	118 (48.2)	127 (51.8)	72 (29.4)	172 (70.2)	7 (2.9)	74 (30.2)	161 (65.7)	NA	NA	3 (1.2)	NA	158.8 (227.23)	NA	Treatment with a once-daily FDC containing netarsudil 0.02% and latanoprost 0.005% targets both the trabecular (conventional) and the uveoscleral outflow pathways, resulting in IOP lowering that is superior to monotherapy with netarsudil or latanoprost. In two phase 3 studies, MERCURY-128 and MERCURY-2, netarsudil/latanoprost FDC was demonstrated to maintain superior IOP lowering over three months, with minimal treatment-related systemic AEs and tolerable ocular safety.
	Netarsudil 0.02%	255	153 (60.0)	64.5 (10.58)	109 (42.7)	146 (57.3)	68 (26.7)	187 (73.3	11 (4.3)	76 (29.8)	165 (64.7)	NA	NA	3 (1.2)	NA	138.7 (197.94)	NA	
	Latanoprost 0.005%	250	144 (57.6)	64.3 (11.41)	112 (44.8)	138 (55.2)	79 (31.6)	171 (68.4)	6 (2.4)	79 (31.6)	163 (65.2)	NA	NA	2 (0.8)	NA	146.0 (211.98)	NA	
Brubaker, 2020 [20]	Netarsudil 0.02%/ Latanoprost 0.005% Fixed-Dose Combination	238	134 (56.3)	64.4 ( 11.33)	109 (45.8) )	129 (54.2)	65 (27.3)	173 (72.7)	7 (2.9)	69 (29.0)	162 (68.1)	NA	0	0	0	403.6 (451.25)	NA	The conclusions are limited by the 12-month duration, given that OAG and OHT are chronic diseases. Longer-term follow-up data on netarsudil/latanoprost FDC are needed to characterize its clinical profile morefully. Nonetheless, it is reassuring that these 12-month data showed IOP-lowering efficacy consistent with the previous three-month analysis.
	Netarsudil 0.02%	244	136 (55.7)	64.6 (10.97)	107 (43.9)	137 (56.1)	57 (23.4)	187 (76.6) )	6 (2.5)	70 (28.7)	167 (68.4)	NA	0	0	1 (0.4)	335.4 (349.28)	NA	
	Latanoprost 0.005%	236	136 (57.6)	65.4 (10.98)	95 (40.3)	141 (59.7)	55 (23.3)	181 (76.7)	10 (4.2)	67 (28.4)	157 (66.5)	NA	0	0	2 (0.8)	336.2 (356.84)	NA	

All of the included population aged more than 60 years. The majority of the included population were females. The patients diagnosed with open-angle glaucoma were almost double of the patients that were diagnosed with ocular hypertension. No differences related to race were detected, and all the conclusions of the included papers were mentioned in the table.

ROB Assessment

**Figure 2:**
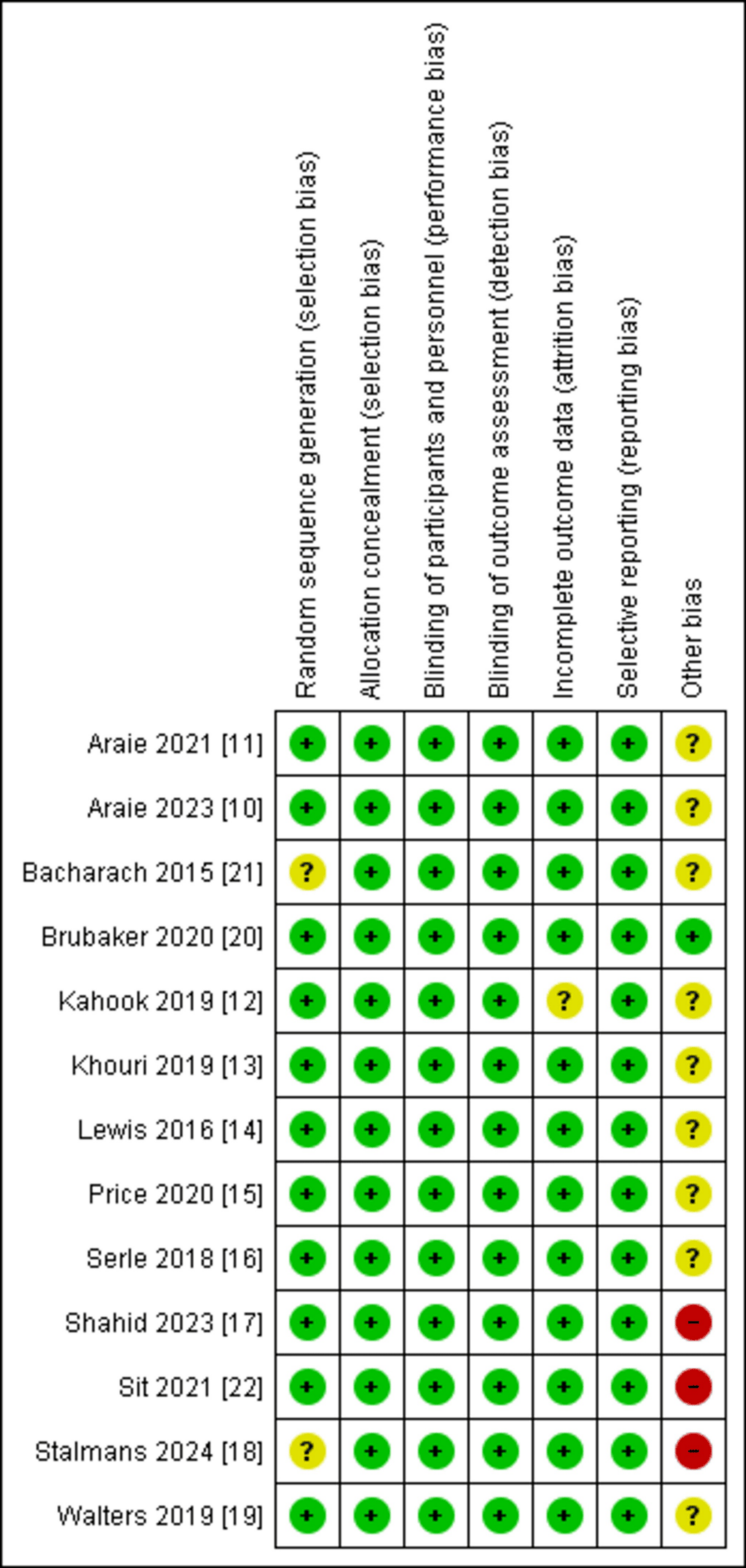
Risk-of-bias summary References: [10-22]

We used the Cochrane ROB 1 tool to estimate the ROB, which showed an overall high quality of the papers included. Figure 2 shows the summary of the ROB in RCTs.

Efficacy outcomes

IOP After One-Week Follow-Up

**Figure 3:**
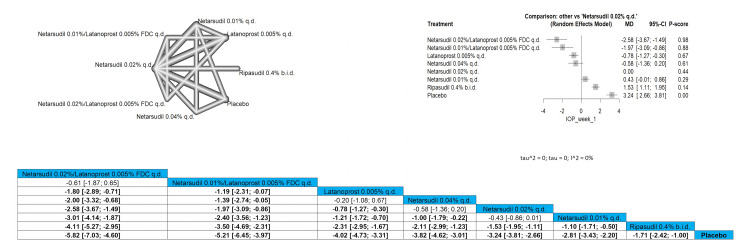
Intraocular pressure (IOP) after one-week follow-up

Netarsudil 0.02%/latanoprost 0.005% FDC q.d. significantly reduced the IOP pressure at the first week when compared to latanoprost 0.005% q.d., netarsudil 0.04% q.d., netarsudil 0.02% q.d., netarsudil 0.01% q.d., ripasudil 0.4% b.i.d, and placebo and results were MD = -1.80 (-2.89; -0.71), MD = -2.00 (-3.32; -0.68), MD = -2.58 (-3.67; -1.49), MD = -3.01 (-4.14; -1.87), MD = -4.11 (-5.27; -2.95), and MD = -5.82 (-7.03; -4.60), respectively. Similar results were obtained when comparing netarsudil 0.01%/latanoprost 0.005% FDC q.d. to the same comparison arms (MD = -1.19 (-2.31; -0.07), MD = -1.39 (-2.74; -0.05), MD = -1.97 (-3.09; -0.86), MD = -2.40 (-3.56; -1.23), MD = -3.50 (-4.69; -2.31), and MD = -5.21 (-6.45; -3.97), respectively). The top three interventions that showed efficacy in reducing the IOP after one week were netarsudil 0.02%/latanoprost 0.005% FDC q.d., followed by netarsudil 0.01%/latanoprost 0.005% FDC q.d. and then latanoprost 0.005% q.d. Full data are shown in Figure 3.

IOP After Two-Week Follow-Up

**Figure 4:**
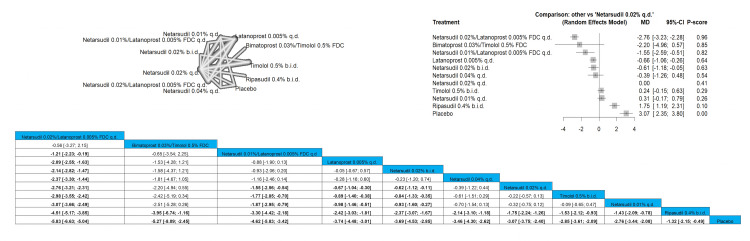
Intraocular pressure (IOP) after two-week follow-up

Netarsudil 0.02%/latanoprost 0.005% FDC q.d. significantly reduced the IOP pressure at two weeks when compared to netarsudil 0.01%/latanoprost 0.005% FDC q.d, latanoprost 0.005% q.d., netarsudil 0.02% b.i.d., netarsudil 0.04% q.d., netarsudil 0.02% q.d., timolol 0.5% b.i.d., netarsudil 0.01% q.d., ripasudil 0.4% b.i.d., and placebo with results of MD = -1.21 (-2.23; -0.19), MD = -2.09 (-2.55; -1.63), MD = -2.14 (-2.82; -1.47), MD = -2.37 (-3.30; -1.44), MD = -2.76 (-3.21; -2.31), MD = -2.98 (-3.55; -2.42), MD = -3.07 (-3.66; -2.49), MD = -4.51 (-5.17; -3.85), MD = -5.83 (-6.63; -5.04)). The top three interventions that showed efficacy in reducing the IOP after two weeks were netarsudil 0.02%/latanoprost 0.005% FDC q.d., followed by bimatoprost 0.03%/timolol 0.5% FDC and then netarsudil 0.01%/latanoprost 0.005% FDC q.d. Full data are shown in Figure 4.

IOP After Four-Week Follow-Up

**Figure 5:**
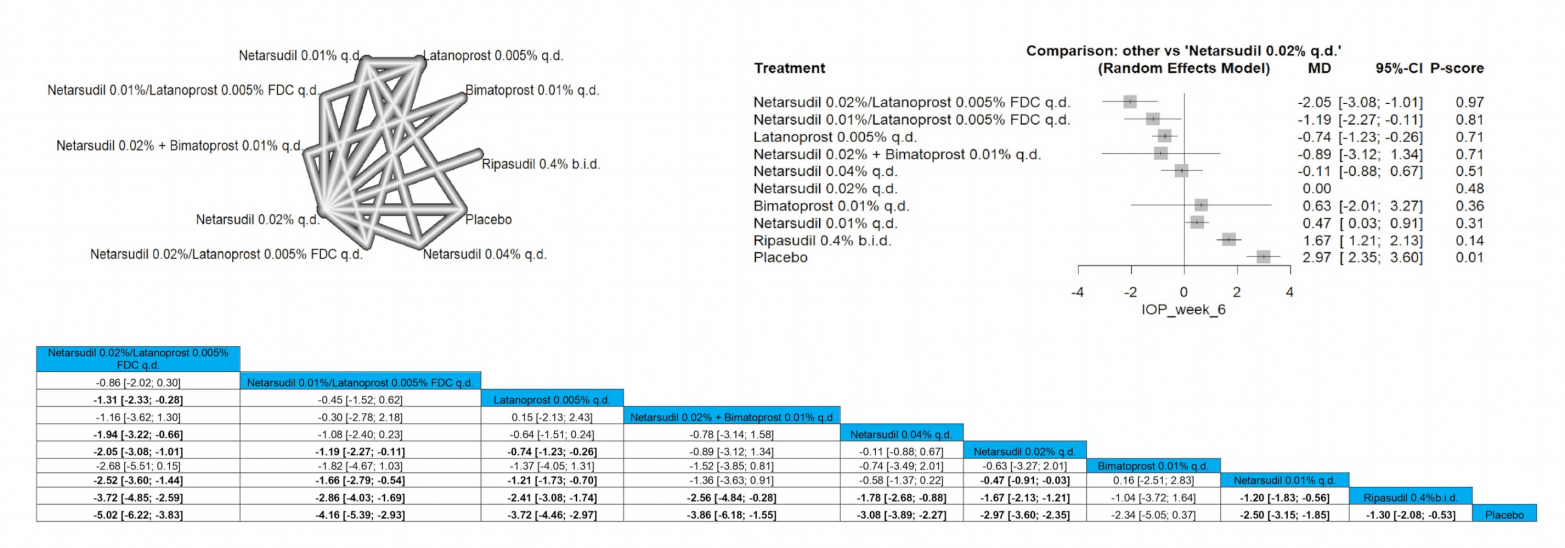
Intraocular pressure (IOP) after four-week follow-up

Netarsudil 0.02%/latanoprost 0.005% FDC q.d. significantly reduced the IOP pressure in four weeks when compared to latanoprost 0.005% q.d. (MD = -1.31 (-2.33; -0.28)). Netarsudil 0.02%/latanoprost 0.005% FDC q.d. also showed significant improvement when compared to netarsudil 0.04% q.d. and netarsudil 0.02% q.d., and the pooled results were (MD = -1.94 (-3.22; -0.66)) and (MD = -2.05 (-3.08; -1.01)). The top three interventions that showed efficacy in reducing the IOP after four weeks were netarsudil 0.02%/latanoprost 0.005% FDC q.d., followed by netarsudil 0.01%/latanoprost 0.005% FDC q.d. and finally latanoprost 0.005% q.d. Full data are shown in Figure 5.

IOP After Six-Week Follow-Up

**Figure 6:**
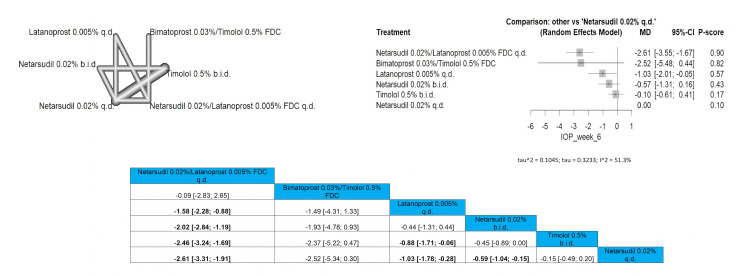
Intraocular pressure (IOP) after six-week follow-up

Only six arms were included in the analysis. Netarsudil 0.02%/latanoprost 0.005% FDC q.d. showed the most superior results when compared to other arms, proving the highest efficacy in this follow-up duration. Netarsudil 0.02%/latanoprost 0.005% FDC q.d. showed a significant reduction in IOP in the six-week time frame when compared to latanoprost 0.005% q.d., netarsudil 0.02% b.i.d., timolol 0.5% b.i.d., and netarsudil 0.02% q.d. ((MD = -1.58 (-2.28; -0.88), MD = -2.02 (-2.84; -1.19), MD = -2.46 (-3.24; -1.69), and MD = -2.61 (-3.31; -1.91), respectively. Full data are shown in Figure 6.

IOP After Three-Month Follow-Up

**Figure 7:**
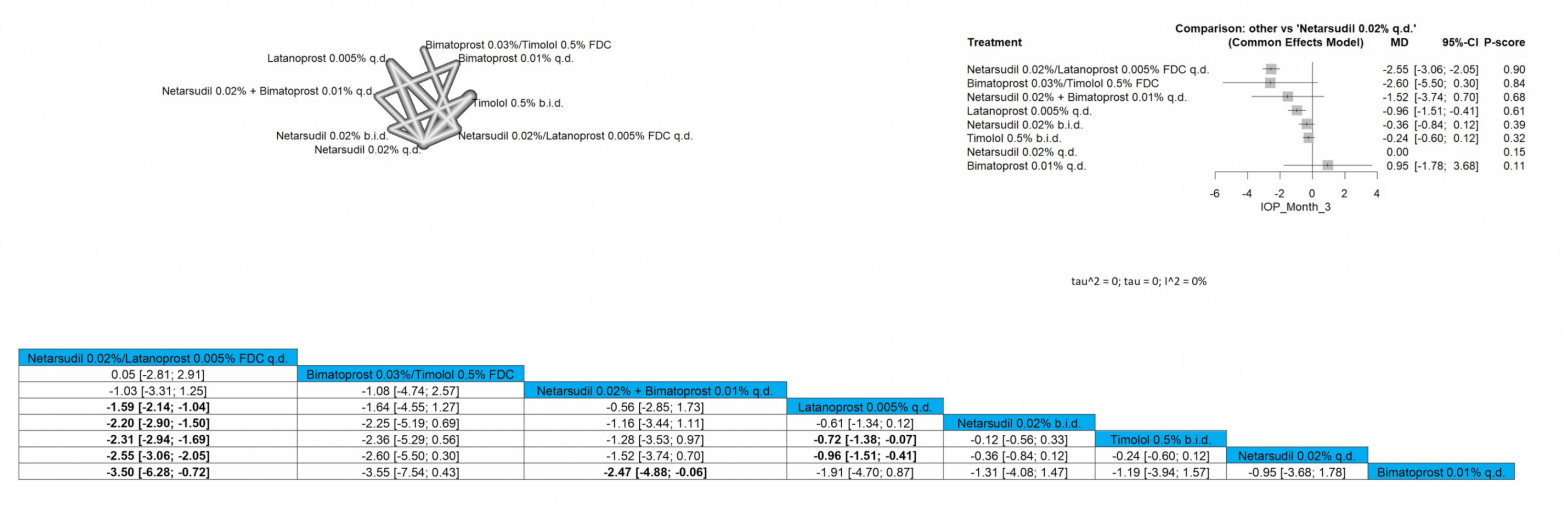
Intraocular pressure (IOP) after three-month follow-up

Eight interventions were compared in this outcome. Netarsudil 0.02%/latanoprost 0.005% FDC q.d. showed the most significant results compared to all other interventions. Netarsudil 0.02%/latanoprost 0.005% FDC q.d. showed a significant reduction in IOP in comparison to all arms except for bimatoprost 0.03%/timolol 0.5% FDC, and netarsudil 0.02% + bimatoprost 0.01% q.d. with pooled results of (MD = 0.05 (-2.81; 2.91)) and (MD = -1.03 (-3.31; 1.25)), respectively. Full data are shown in Figure 7.

Mean Diurnal IOP 14 mmHg or Less

**Figure 8:**
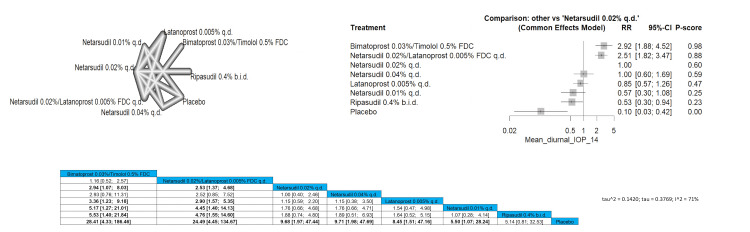
Mean diurnal intraocular pressure (IOP) 14 mmHg or less

Our meta-analysis assessed the proportion of patients achieving a mean diurnal IOP of 14 mmHg or less. The pooled results for this outcome were RR = 2.94 (1.07; 8.03), indicating a significant improvement in IOP control with bimatoprost 0.03%/timolol 0.5% FDC compared to netarsudil 0.02% q.d. Both bimatoprost 0.03%/timolol 0.5% FDC, and netarsudil 0.02%/latanoprost 0.005% FDC q.d. were significantly superior when compared to latanoprost 0.005% q.d., netarsudil 0.01% q.d., ripasudil 0.4% b.i.d., and placebo. Bimatoprost 0.03%/timolol 0.5% FDC and netarsudil 0.02%/latanoprost 0.005% FDC q.d. showed the most superior improvement regarding this outcome. Full data are shown in Figure 8.

Mean Diurnal IOP 14 mmHg or Less

**Figure 9:**
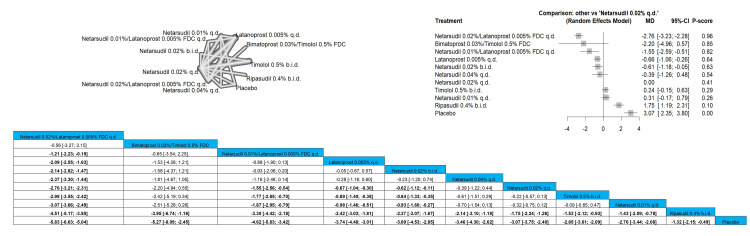
Mean diurnal intraocular pressure (IOP) 15 mmHg or less

Bimatoprost 0.03%/timolol 0.5% FDC was the most superior intervention, followed by netarsudil 0.02%/latanoprost 0.005% FDC q.d. and then netarsudil 0.01%/latanoprost 0.005% FDC q.d., bimatoprost 0.03%/timolol 0.5% FDC and netarsudil 0.02%/latanoprost 0.005% FDC q.d.showed a significant difference with all interventions except for netarsudil 0.01%/latanoprost 0.005% FDC q.d. showing a result of RR = 1.92 (0.82; 4.50) and RR = 1.75 (0.88; 3.48). Full data are shown in Figure 9.

Mean Diurnal IOP 16 mmHg or Less

**Figure 10:**
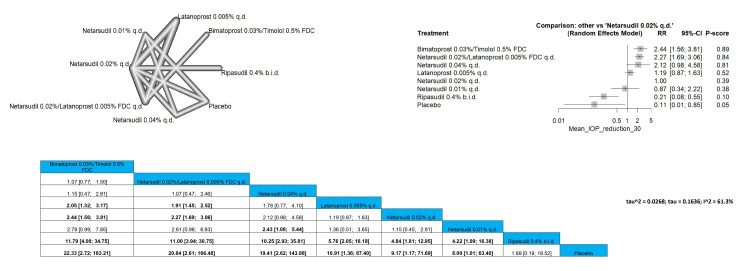
Mean diurnal intraocular pressure (IOP) 16 mmHg or less

The results showed that bimatoprost 0.03%/timolol 0.5% FDC is the most superior intervention, followed by netarsudil 0.02%/latanoprost 0.005% FDC q.d. and then netarsudil 0.01%/latanoprost 0.005% FDC q.d. These three interventions were significant with all other interventions and showed no significance compared to others. Full data are shown in Figure 10.

Mean Diurnal IOP 17 mmHg or Less

**Figure 11:**
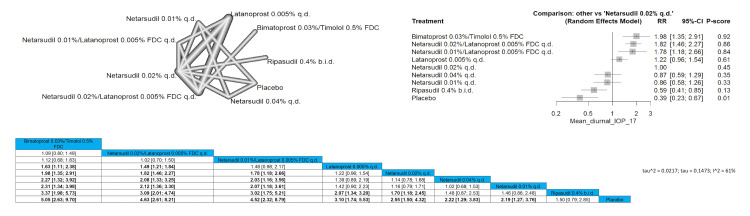
Mean diurnal intraocular pressure (IOP) 17 mmHg or less

Bimatoprost 0.03%/timolol 0.5% FDC showed significant improvement in the IOP reduction compared to all other arms except for netarsudil 0.02%/latanoprost 0.005% FDC q.d. and netarsudil 0.01%/latanoprost 0.005% FDC q.d. with pooled results of (RR = 1.09 (0.80; 1.49), (RR = 1.12 (0.68; 1.83)), respectively. The results showed that bimatoprost 0.03%/timolol 0.5% FDC is the most superior intervention regarding this outcome, followed by netarsudil 0.02%/latanoprost 0.005% FDC q.d. Full data are shown in Figure 11.

Mean Diurnal IOP 18 mmHg or Less

**Figure 12:**
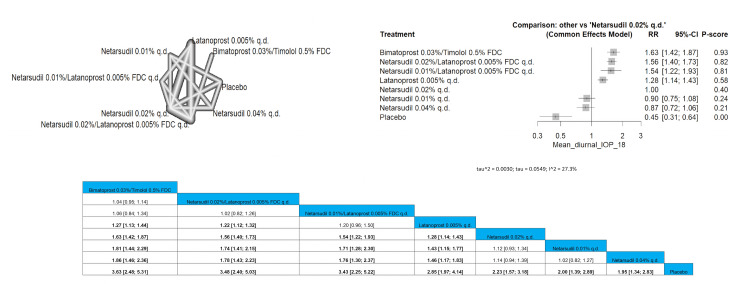
Mean diurnal intraocular pressure (IOP) 18 mmHg or less

Bimatoprost 0.03%/timolol 0.5% FDC showed significant improvement in the IOP reduction compared to all other arms except for netarsudil 0.02%/latanoprost 0.005% FDC q.d. and netarsudil 0.01%/latanoprost 0.005% FDC q.d. with pooled results of (RR = 1.04 (0.95; 1.14), (RR = 1.06 (0.84; 1.34)), respectively. The results showed that bimatoprost 0.03%/timolol 0.5% FDC is the most superior intervention regarding this outcome, followed by netarsudil 0.02%/latanoprost 0.005% FDC q.d. and then netarsudil 0.01%/latanoprost 0.005% FDC q.d. Full data are shown in Figure 12.

Mean IOP Reduction of 20% or More

**Figure 13:**
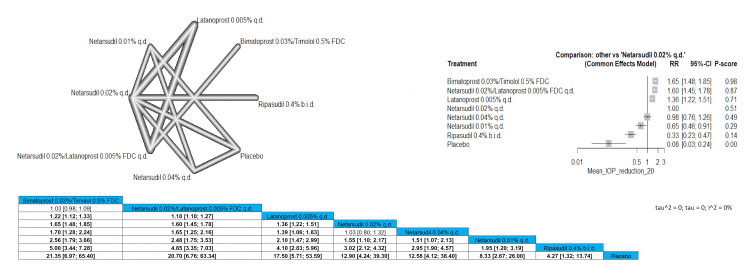
Mean intraocular pressure (IOP) reduction of 20% or more

Bimatoprost 0.03%/timolol 0.5% FDC was significantly effective in the reduction of the IOP compared to all other arms except for netarsudil 0.02%/latanoprost 0.005% FDC q.d. with pooled results of RR = 1.03 (0.98; 1.09). According to our analysis, ripasudil 0.4% b.i.d. was the least effective medication to be used in the outcome although it also proved to be significantly better than placebo (RR = 4.27 (1.32; 13.74)). The most superior arm in achieving 20% or less reduction in the IOP is bimatoprost 0.03%/timolol 0.5% FDC followed by netarsudil 0.02%/latanoprost 0.005% FDC q.d. and then latanoprost 0.005% q.d. Full data are shown in Figure 13.

Mean IOP Reduction of 25% or More

**Figure 14:**
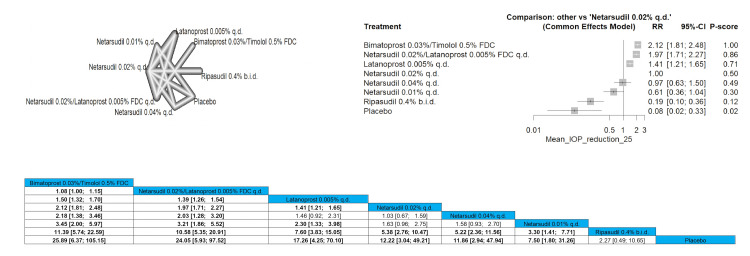
Mean IOP reduction of 25% or more

Similar to the previous outcome the bimatoprost 0.03%/timolol 0.5% FDC was the most effective in reducing the IOP as it showed a significant reduction compared to all other arms except for netarsudil 0.02%/latanoprost 0.005% FDC q.d. that showed a barely significant difference with pooled results of RR = 1.08 (1.00; 1.15). The most superior intervention according to P-score in the outcome is bimatoprost 0.03%/timolol 0.5% FDC, followed by netarsudil 0.02%/latanoprost 0.005% FDC q.d. Full data are shown in Figure 14.

Mean IOP Reduction of 30% or More

**Figure 15:**
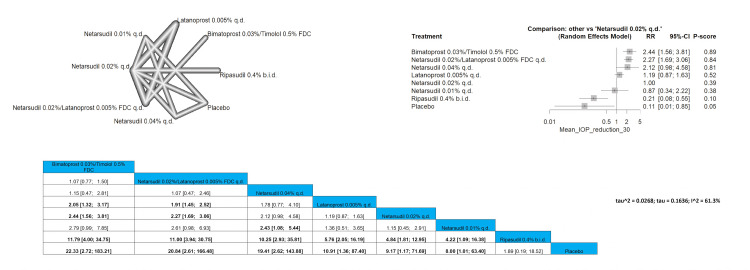
Mean intraocular pressure (IOP) reduction of 30% or more

Bimatoprost 0.03%/timolol 0.5% FDC showed a significantly higher number of patients achieving the 30% IOP reduction when compared to latanoprost 0.005% q.d., netarsudil 0.02% q.d., ripasudil 0.4% b.i.d., and placebo, and the pooled results were RR = 2.05 (1.32; 3.17), RR = 2.44 (1.56; 3.81), RR = 11.79 (4.00; 34.75), and RR = 22.33 (2.72; 183.21), respectively. The same results were obtained when netarsudil 0.02%/latanoprost 0.005% FDC q.d. was compared to the same arms. The most superior intervention according to the P-score in the outcome is bimatoprost 0.03%/timolol 0.5% FDC, followed by netarsudil 0.02%/latanoprost 0.005% FDC q.d. and then netarsudil 0.04% q.d. Full data are shown in Figure 15.

Mean IOP Reduction of 35% or More

**Figure 16:**
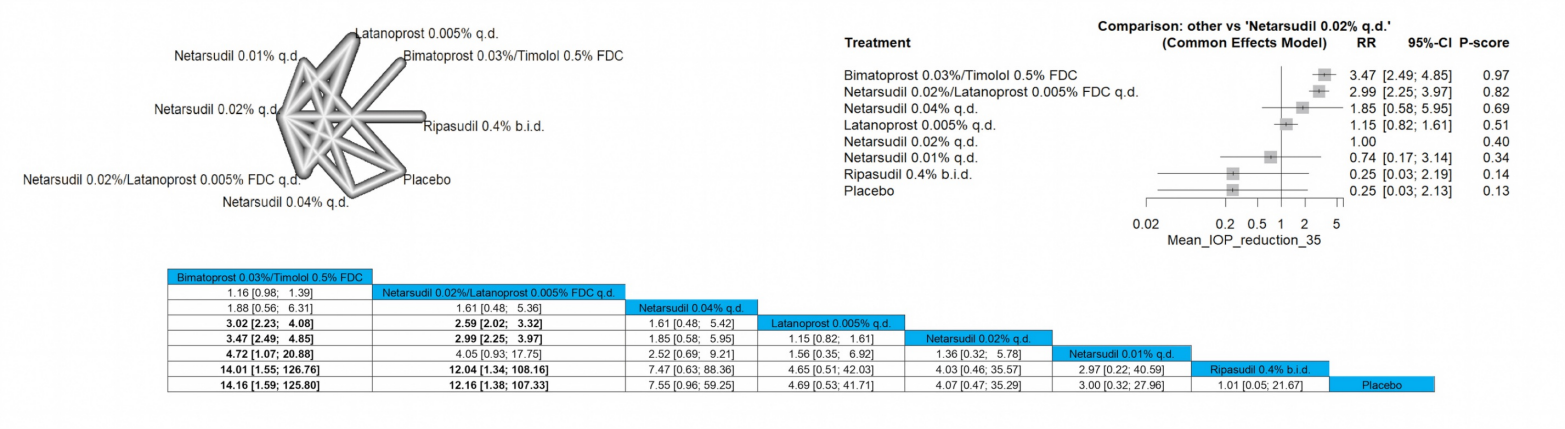
Mean intraocular pressure (IOP) reduction of 35% or more

Bimatoprost 0.03%/timolol 0.5% FDC was significant in achieving the 35% reduction of IOP with all the compared arms except for netarsudil 0.02%/latanoprost 0.005% FDC q.d. and netarsudil 0.02% q.d. with pooled results of RR = 1.16 (0.98; 1.39) and RR = 1.88 (0.56; 6.31), respectively. The most superior intervention according to the P-score in the outcome is bimatoprost 0.03%/timolol 0.5% FDC, followed by netarsudil 0.02%/latanoprost 0.005% FDC q.d. Full data are shown in Figure 16.

Mean IOP Reduction of 40% or More

**Figure 17:**
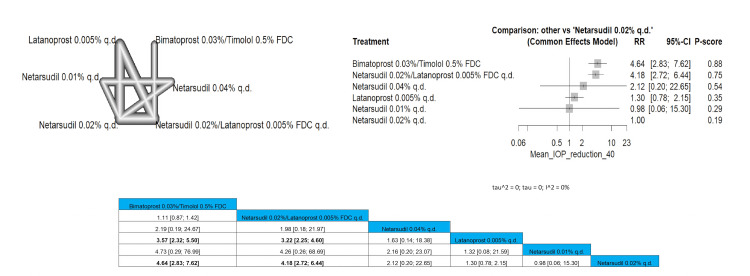
Mean intraocular pressure (IOP) reduction of 40% or more

The patients that achieved 40% mean IOP reduction were found mostly in the bimatoprost 0.03%/timolol 0.5% FDC arm that showed significance when compared to latanoprost 0.005% q.d. and placebo (RR =3.57 (2.32; 5.50) and RR = 4.64 (2.83; 7.62)). Similarly, netarsudil 0.02%/latanoprost 0.005% FDC q.d. was significant with the same arms providing results of RR = 3.22 (2.25; 4.60) and RR = 4.18 (2.72; 6.44). Full data are shown in Figure 17.

Safety outcomes

Blurred Vision

**Figure 18:**
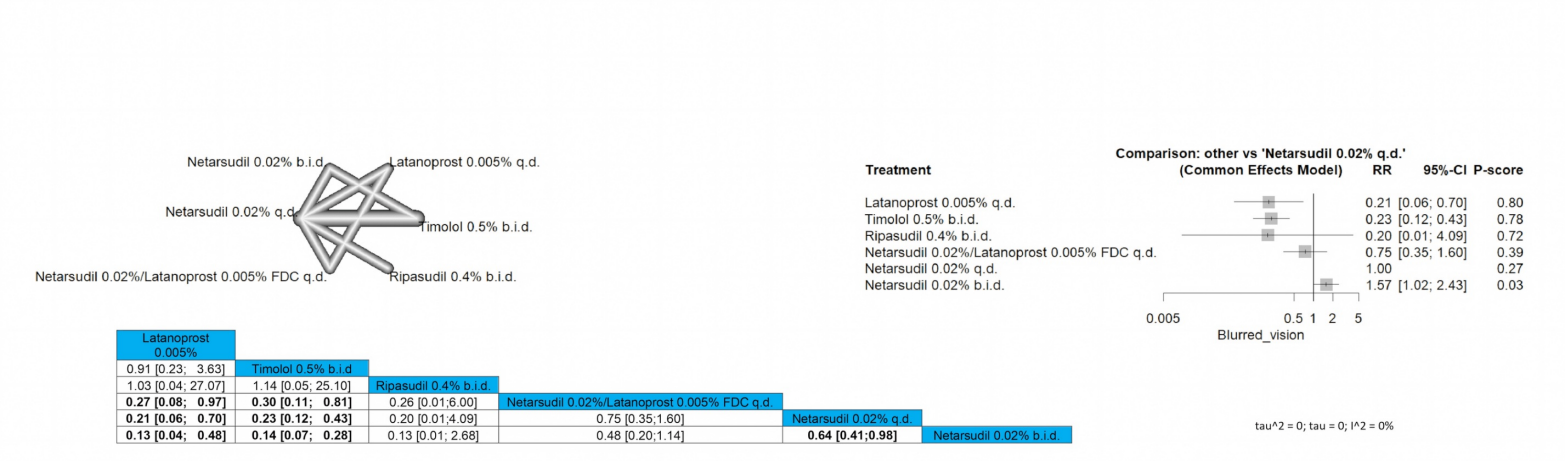
Blurred vision

The results showed a significantly lower incidence of blurred vision when comparing latanoprost 0.005% to netarsudil 0.02%/latanoprost 0.005% FDC q.d., netarsudil 0.02% q.d, and netarsudil 0.02% b.i.d. (RR = 0.27 (0.08; 0.97), 0.21 (0.06; 0.70), and 0.13 (0.04; 0.48), respectively). The results showed a significant reduction of blurred vision incidence when comparing timolol 0.5% b.i.d to netarsudil 0.02%/latanoprost 0.005% FDC q.d., netarsudil 0.02% q.d., and netarsudil 0.02% b.i.d. (RR = 0.30 (0.11; 0.81), RR = 0.23 (0.12; 0.43), and RR = 0.14 (0.07; 0.28), respectively). Netarsudil 0.02% q.d. showed a significantly lower incidence of blurred vision when compared to netarsudil 0.02% b.i.d. (RR = 0.64 (0.41; 0.98)). The order of drugs in terms of the lowest incidence of blurred vision is latanoprost 0.005%, followed by timolol 0.5% b.i.d. Full data are shown in Figure 18.

Conjunctival Hemorrhage

**Figure 19:**
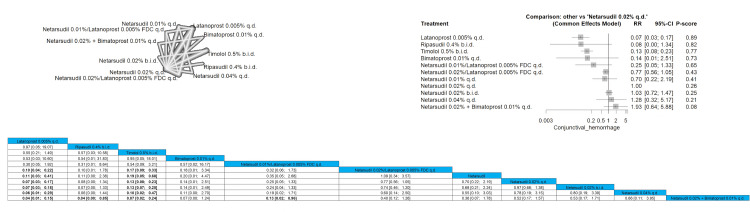
Conjunctival hemorrhage

The results showed a significantly lower incidence of conjunctival hemorrhage when comparing latanoprost 0.005% to netarsudil 0.02%/latanoprost 0.005% FDC q.d., netarsudil 0.02% q.d., netarsudil 0.02% b.i.d., netarsudil 0.04% q.d., and netarsudil 0.02% + Bimatoprost 0.01% q.d., and the results were RR = 0.10, RR = 0.11, RR = 0.07, RR = 0.06, and RR = 0.04, respectively. The same results were obtained when comparing timolol 0.5% b.i.d. to latanoprost 0.005% to netarsudil 0.02%/latanoprost 0.005% FDC q.d., netarsudil 0.02% q.d., netarsudil 0.02% b.i.d., netarsudil 0.04% q.d., and netarsudil 0.02% + bimatoprost 0.01% q.d. (RR = 0.17, RR = 0.19, RR = 0.13, RR = 0.10, and RR = 0.07). The order of drugs in terms of the lowest incidence of conjunctival hemorrhage is latanoprost 0.005% q.d., followed by ripasudil 0.4% b.i.d. and then timolol 0.5% b.i.d. Full data are shown in Figure 19.

Conjunctival Hyperemia

**Figure 20:**
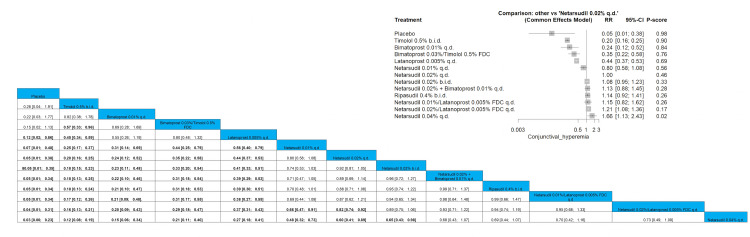
Conjunctival hyperemia

The results showed a significantly lower incidence of conjunctival hyperemia when comparing timolol 0.5% b.i.d. to all other arms except for placebo and bimatoprost 0.01% q.d. It should be noted that bimatoprost 0.03%/timolol 0.5% FDC showed a significantly lower incidence of this negative outcome in comparison to netarsudil 0.02%/latanoprost 0.005% FDC q.d. with pooled results of RR = 0.29 (0.18; 0.47). The order of drugs in terms of the lowest incidence of conjunctival hyperemia is placebo, followed by timolol 0.5% b.i.d. and then bimatoprost 0.01% q.d. Full data are shown in Figure 20. 

Cornea Verticillata

**Figure 21:**
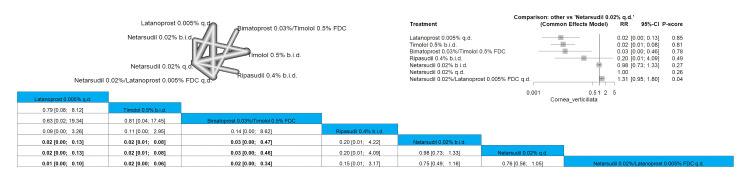
Cornea verticillata

The results showed that latanoprost 0.005% q.d., timolol 0.5% b.i.d., and bimatoprost 0.03%/timolol 0.5% FDC showed a significantly lower incidence of cornea verticillata compared to the other arms. Latanoprost 0.005% q.d. has a significantly lower incidence of cornea verticillata when compared to netarsudil 0.02% b.i.d., netarsudil 0.02% q.d., and netarsudil 0.02%/latanoprost 0.005% FDC q.d., and the results were RR = 0.02 (0.00; 0.13), RR = 0.02 (0.00; 0.13), and RR = 0.01 (0.00; 0.10), respectively. It should be noted that bimatoprost 0.03%/timolol 0.5% FDC showed a significantly lower incidence of this negative outcome than netarsudil 0.02%/latanoprost 0.005% FDC q.d. with pooled results of RR = 0.02 (0.00; 0.34). Full data are shown in Figure 21.

Eye Pruritus

**Figure 22:**
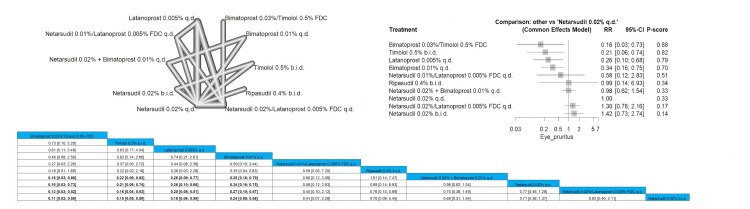
Eye pruritus

Bimatoprost 0.03%/timolol 0.5% FDC had significantly lower incidence of eye pruritus in comparison to netarsudil 0.02% + bimatoprost 0.01% q.d., netarsudil 0.02% q.d., netarsudil 0.02%/latanoprost 0.005% FDC q.d., and netarsudil 0.02% b.i.d., and the results were RR = 0.16 (0.03; 0.80), RR = 0.16 (0.03; 0.73), RR = 0.12 (0.03; 0.52), and RR = 0.11 (0.02; 0.59), respectively. The order of drugs in terms of the lowest incidence of eye pruritus was bimatoprost 0.03%/timolol 0.5% FDC, followed by timolol 0.5% b.i.d. and then latanoprost 0.005% q.d. Full data are shown in Figure 22.

Eye Irritation

**Figure 23:**
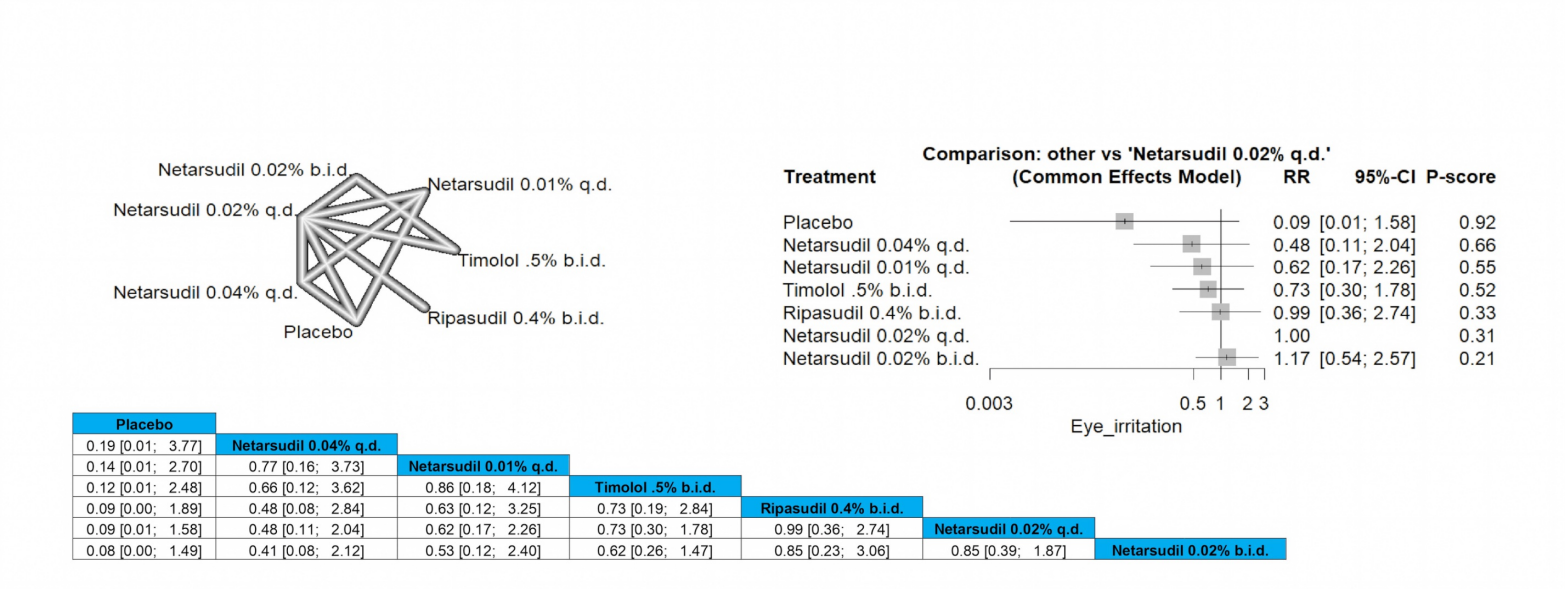
Eye irritation

No significant difference in the incidence of eye irritation was found as all the interventions were similar in the results. Full data are shown in Figure 23.

Discussion

Regarding IOP reduction in this study, the combination of netarsudil 0.02%/latanoprost 0.005% fixed-dose combination (FDC) once daily is the most superior intervention achieving a significant difference in different outcomes at various follow-up durations when compared to other interventions. Similarly, bimatoprost 0.03%/timolol 0.5% FDC and netarsudil 0.02%/latanoprost 0.005% FDC once daily showed the most superior improvement in the mean diurnal IOP.

The most frequently reported ocular adverse events were conjunctival hyperemia, conjunctival hemorrhage, and cornea verticillata. The highest incidence of conjunctival hyperemia was reported among patients who were treated with netarsudil and netarsudil combination therapies. On the other hand, the patients who were treated with timolol 0.5% twice daily showed the lowest incidence of conjunctival hyperemia. Furthermore, no serious systemic or ocular adverse events were reported. The lowest incidence of conjunctival hemorrhage is latanoprost 0.005% once daily, followed by ripasudil 0.4% twice daily and then timolol 0.5% twice daily. No significant difference was found between the arms regarding the eye irritation side effect.

The main cause of the observed conjunctival hyperemia is the Rho kinase inhibitor action that induces vasodilation by relaxing vascular smooth muscles [23]. This adverse event could worsen and could have an impact on the quality of life for the patient resulting in discomfort and affecting the visual function. We noted from our analysis that all netarsudil combination drugs were associated with a higher incidence of conjunctival hyperemia; we also found that bimatoprost 0.03%/timolol 0.5% FDC was significantly lower in incidence of conjunctival hyperemia compared to all the arms including netarsudil.

Corneal verticillata is characterized by distinctive concentric, whorl-like patterns on the cornea. This finding could only be detected using a biomicroscope and did not affect visual function during the treatment [16]. Cornea verticillata is spontaneously resolved in most patients. Multiple cationic and amphiphilic drugs such as netarsudil induce cornea verticillata due to a process called phospholipidosis. It is a lysosomal accumulation of phospholipids within corneal epithelial cells [24]. Corneal verticillata has been associated with various agents, including nonsteroidal anti-inflammatory drugs, systemic amiodarone, and ocular medications like tobramycin and gentamicin [25]. It is rare for cornea verticillata to affect visual acuity or to cause ocular symptoms. In addition, it is typically resolved with the discontinuation of the drug [26]. According to our results, the combinations of the drugs resulted in a significant increase in the incidence of corneal verticillata compared to monotherapy. On the other hand, the combination of bimatoprost 0.03%/timolol 0.5% FDC showed significantly lower incidence and high effectiveness. Bimatoprost 0.03%/timolol 0.5% showed even a significant difference when compared to netarsudil 0.02%/latanoprost 0.005% FDC q.d.

Rho kinase inhibitors are a novel class of antiglaucoma medications that act on the Rho-associated protein kinase (ROCK) pathway and norepinephrine transporter. These drugs decrease the IOP by three different mechanisms: increased trabecular outflow, reduced episcleral venous pressure (EVP), and decreased aqueous humor production [27,28]. Netarsudil ophthalmic solution (0.02%) is a therapeutic agent that is classified as a Rho kinase inhibitor. It was approved by the FDA in 2017 to treat glaucoma and ocular hypertension. The FDA approved netarsudil based on the ROCKET-1, ROCKET-2, and ROCKET-4 trials [29]. These phase 3 clinical studies assessed the efficacy and safety of netarsudil ophthalmic solution (0.02%) in reducing IOP in patients with open-angle glaucoma or ocular hypertension.

In ROCKET-1, the primary efficacy analysis focused on patients with baseline IOP < 27 mmHg, with a secondary post-hoc analysis for those <25 mm Hg. Both netarsudil (0.02%) once daily and timolol showed comparable IOP reductions, with baseline IOPs decreasing from approximately 21.5-23.4 mm Hg to 17.2-19.8 mm Hg for netarsudil and 17.4-18.5 mm Hg for timolol. The mean IOP reduction ranged from 15%-22% for netarsudil and 17%-22% for timolol; however, netarsudil did not meet the criteria for noninferiority to timolol [13].

In the ROCKET-2 study, patients with baseline IOP < 25 mm Hg, netarsudil (both once and twice daily), and timolol showed similar baseline IOP reductions over three months follow-up. The mean IOP decreased to 16.7-18.2 mmHg for netarsudil once daily, 15.7-17.6 mmHg for netarsudil twice daily, and 16.6-17.7 mmHg for timolol, with reductions from baseline of 16-21% for netarsudil once daily, 22-24% for netarsudil twice daily, and 18-23% for timolol. Both dosing regimens of netarsudil met the noninferiority criteria compared to timolol [16].

ROCKET-4 established the effectiveness of once-daily netarsudil compared to twice-daily timolol. It also demonstrated non-inferiority at predetermined IOP ranges. All studies consistently showed IOP reduction across different baseline levels during the 90-day assessment [17,29].

The Rho kinase inhibitors may provide an additional IOP-lowering effect when used in combination with other glaucoma medications [28]. A previous study (Shahid et al.) demonstrated that combination therapy of netarsudil 0.02% and bimatoprost 0.01% achieved a maximum IOP reduction of 44% compared to either agent used alone, which aligns with our findings in the study as this combination resulted in significant reduction in comparison to other arms [17].

This study has several strength points, including a larger number of studies and a large number of populations, having a high overall quality of the included studies, including all the covered outcomes to provide a comprehensive evaluation of the outcomes and a more extensive exploration of various drugs and combinations to give a recommendation for the best treatment option in case of elevated IOP patients. We also covered various follow-up durations to evaluate the short-term and long-term effectiveness. We evaluated all the possible outcomes related to all available drugs whether in single therapy or in combination. We also had some limitations as the studies had some heterogeneity and differences, and the safety outcomes were estimated at different follow-up durations.

## Conclusions

The analysis reveals that while netarsudil 0.02%/latanoprost 0.005% FDC once daily emerged as the most effective intervention for IOP reduction, followed by bimatoprost 0.03%/timolol 0.5% FDC, careful consideration of the safety profile is essential for treatment selection. The main adverse events observed were conjunctival hyperemia (most frequent with netarsudil combinations), conjunctival hemorrhage (lowest with latanoprost 0.005%), and cornea verticillata, although no serious systemic or ocular adverse events were reported. Notably, while netarsudil combinations demonstrated superior IOP reduction through multiple mechanisms, bimatoprost 0.03%/timolol 0.5% FDC presented a compelling alternative with better tolerability while maintaining high effectiveness. This comprehensive analysis, supported by a large study population and high-quality data across various follow-up durations, suggests that clinicians should carefully balance treatment efficacy with potential adverse events, particularly considering patient-specific factors such as tolerance for cosmetic side effects that might impact treatment compliance.
